# Genomic Analysis of *Kitasatospora setae* to Explore Its Biosynthetic Potential Regarding Secondary Metabolites

**DOI:** 10.3390/antibiotics13050459

**Published:** 2024-05-16

**Authors:** Yutong Xue, Zhiyan Zhou, Fangjian Feng, Hang Zhao, Shuangling Tan, Jinling Li, Sitong Wu, Zhiran Ju, Shan He, Lijian Ding

**Affiliations:** 1 Li Dak Sum Yip Yio Chin Kenneth Li Marine Biopharmaceutical Research Center, Ningbo University, Ningbo 315211, China; xyt1226@outlook.com (Y.X.); 2211390043@nbu.edu.cn (F.F.); zhaohang156568@163.com (H.Z.); tslidid@outlook.com (S.T.); ljl464400@163.com (J.L.); 2 School of Pharmacy, Ningbo University, Ningbo 315211, China; 18357051305@163.com; 3 School of Pharmaceutical Sciences, Zhejiang University of Technology, Hangzhou 310014, China; 202105530218@zjut.edu.cn (S.W.); zhiranju0409@zjut.edu.cn (Z.J.)

**Keywords:** rare actinomycete, *Kitasatospora*, genome, antiSMASH analysis, GNPS

## Abstract

Actinomycetes have long been recognized as important sources of clinical antibiotics. However, the exploration of rare actinomycetes, despite their potential for producing bioactive molecules, has remained relatively limited compared to the extensively studied *Streptomyces* genus. The extensive investigation of *Streptomyces* species and their natural products has led to a diminished probability of discovering novel bioactive compounds from this group. Consequently, our research focus has shifted towards less explored actinomycetes, beyond *Streptomyces*, with particular emphasis on *Kitasatospora setae* (*K. setae*). The genome of *K. setae* was annotated and analyzed through whole-genome sequencing using multiple bio-informatics tools, revealing an 8.6 Mbp genome with a 74.42% G + C content. AntiSMASH analysis identified 40 putative biosynthetic gene clusters (BGCs), approximately half of which were recessive and unknown. Additionally, metabolomic mining utilizing mass spectrometry demonstrated the potential for this rare actinomycete to generate numerous bioactive compounds such as glycosides and macrolides, with bafilomycin being the major compound produced. Collectively, genomics- and metabolomics-based techniques confirmed *K. setae*’s potential as a bioactive secondary metabolite producer that is worthy of further exploration.

## 1. Introduction

In recent years, the issue of antimicrobial resistance (AMR) has emerged as a formidable global challenge. Consequently, there is an increasing imperative to discover antibiotics with novel structures or mechanisms of action in order to effectively combat this problem [1]. Natural products have been and continue to be pivotal and irreplaceable in the development of new pharmaceuticals. Actinomycetes, which are renowned for their production of biologically active natural compounds [2], are responsible for synthesizing about two-thirds of clinically used antibiotics and also contribute to a diverse array of anticancer agents and immunosuppressive compounds [3,4]. Approximately 70% of antibiotics are derived from the genus *Streptomyces*, which includes streptomycin, oxytetracycline, and jinggangmycin (a product of *Streptomyces jinggangensis* from China) [5]. However, the potential of rare actinomycetes to produce bioactive natural products remains unclear due to their relatively limited isolation under standard laboratory culture conditions. Therefore, conducting comprehensive research on untapped actinomycete resources and their bioactive secondary metabolites holds significant scientific value in the pursuit of novel antibiotics and anticancer or antiviral drugs [6].

The traditional drug discovery paradigm, which primarily focuses on bioactivity, has frequently resulted in the rediscovery of numerous known compounds [7]. Therefore, it is crucial to incorporate advanced techniques such as ‘molecular networking dereplication’ and ‘multi-omics directional mining’. Molecular networking, generated through an algorithm that compares similarities between fragment spectra in LC-MS/MS data, facilitates the identification of chemical diversity and aids in determining compound structures [8]. Structurally related compounds can be clustered together with analogues due to their similar MS/MS fragmentation spectra. Dereplication is often performed using large spectrometric databases as a reference, significantly expediting the discovery of novel natural products [9]. The BGC within actinomycetes genomes determines their capacity for antibiotic production, whereas the associated metabolome is the realization of that potential. Modern ‘omics’ technologies have revealed that the genomic potential of the actinomycetes extends far beyond the characterized chemical space. That the biosynthetic capacity of actinomycetes is significantly greater than what is observed under laboratory conditions may be attributed to the down-regulation or silencing of biosynthetic genes. To investigate secondary metabolites in silenced or down-regulated genomes, the introduction of specific stimuli may be required [10,11,12]. Furthermore, advanced experimental protocols such as genomic and metabolomic analyses play increasingly important roles in identifying strains and biosynthetic genes that are likely to produce the desired bioactive molecules.

Rare actinomycetes are currently gaining increased attention in research aiming to discover structurally novel bioactive natural products, since these organisms inhabit unexplored ecological niches, and may thereby reduce the likelihood of compound rediscovery [13]. Advances in genetic sequencing technology and bioinformatics tools have greatly accelerated the discovery and analysis of secondary metabolic gene clusters and their products, while also significantly reducing the associated financial costs. The diversity of biosynthetic secondary metabolite gene clusters (BGCs) that can be identified through genomic sequencing provides crucial insights for the efficient exploration of new natural products [14]. Previous studies have demonstrated that members of the genus *Streptomyces* typically possess 20–50 BGC, which is a substantial number compared to what other actinomycetes tend to have [15]. In addition, certain rare genera of actinomycetes such as *Kitasatospora*, *Saccharothrix*, *Catenulispora*, and *Kibdelosporangium* were found to have a similar number of clusters or slightly fewer gene clusters than *Streptomyces* [14]. Notably, investigations have revealed the presence of numerous biologically active compounds in *Kitasatospora*, and related genomic information (e.g., for *Kitasatospora* sp. MMS16-BH015) contains diverse genes responsible for secondary metabolite biosynthesis, antibiotic resistance, and multi-metal resistance [16]. Additionally, four unique cyclic peptides, known as zelkovamycins, were isolated from the endophyte *Kitasatospora* sp. and exhibited potent inhibitory activity against influenza A (H1N1) virus.

Insufficient research has been conducted on the genomes and metabolomes of rare actinomycetes, leading to relatively limited knowledge about the natural products that they biosynthesize. Therefore, it is imperative to enhance research efforts regarding the natural products of rare actinomycetes and to use bioinformatics tools to investigate them. This study explores the genomics and metabolomics of *Kitasatospora setae*, a less-studied, rare actinomycete species known for its abundance of BGCs, in order to demonstrate and describe its potential for the production of natural products.

## 2. Results

### 2.1. Genome Profile of the Strain K. setae

**Figure 1:**
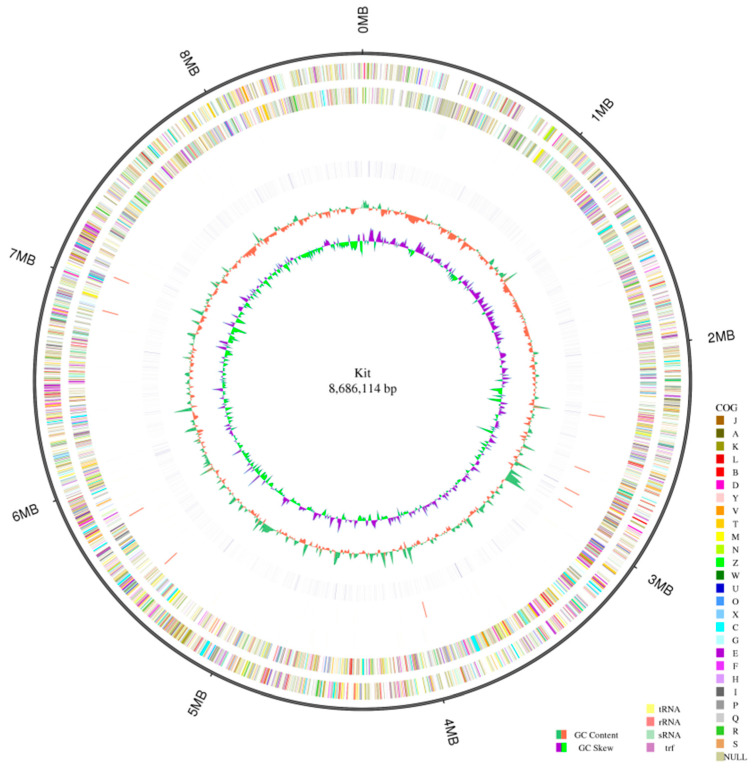
Circular genome map of the strain *K. setae* (the following elements are shown in order from the inner layer to the outer layer: GC-SKEW, GC content, repeat, Reverse-Strand ncRNA, Forward-Strand ncRNA, Reverse-Strand Gene (colored according to the cluster of orthologous groups’ classification), Forward-Strand Gene (colored according to the cluster of orthologous groups’ classification), and genome size.).

Third-generation genome sequencing was performed on strains of *K. setae* using the Nanopore platform. A GC skew analysis was conducted of the sequenced bacterial genome using the (G − C)/(G + C) calculation method. The distribution of each element, such as gene distribution, ncRNA distribution, and annotation data, was displayed on the genome. The results revealed that the genome of *K. setae* consisted of 8.6 Mbp, with a total gene length of 7,552,416 bp (Figure 1). Furthermore, the high GC content of 74.42% in this strain indicates that the DNA of these strains is highly heat-stable, which poses challenges for sequencing assembly and primer design (Table S1). The sequencing results demonstrated that most of the genes in this lineage were less than 1000 nt in length, with a small number of genes ranging between 1000 and 2000 nt, and only a small proportion of the genes exceeded 2000 nt in length (Figure S1).

The whole genome of *K. setae* was predicted to encompass 72 tRNA genes, 27 rRNA genes, and 65 sRNA genes (Table S2) through sequencing. Additionally, the results of prophage gene sequence prediction and comparison indicated that the *K. setae* strain possesses 20 potential prophage genes. The presence of these prophage sequences may facilitate the acquisition of antibiotic resistance in *K. setae*, enhancing its environmental adaptability.

### 2.2. Genome Annotation of K. setae

To obtain functional annotations of gene models in strain *K. setae*, putative protein-coding sequences were analyzed using the BLAST search function in the Cluster of Orthologous Groups (COG) of proteins, Kyoto Encyclopedia of Genes and Genomes (KEGG), and Gene Ontology (GO) databases. Based on the GO database annotations, we can categorize genes into different broad categories to determine their possible functions.

The GO database revealed a total of 3949 annotated genes, accounting for 48.91% of all genes, and these were distributed across three main categories (cellular component, molecular function, and biological process) and 30 subcategories (Figure S2). The majority of genes were associated with ‘catalytic process’, followed by ‘metabolic process’, ‘cellular process’, and ‘binding’. To further understand the genetic functions of the strains of *K. setae*, 3429 (42.47%) genes were annotated and assigned to 43 different KEGG pathways. The KEGG pathway annotation was divided into six categories: cellular processes, environment, genetics, human diseases, metabolism, and organism systems. With these database annotations, it became straightforward to identify all the genes included in the annotations that were associated with a specific type of function. Most proteins were classified into the metabolism panel, specifically the ‘carbohydrate metabolism’ and ‘amino acid metabolism’ pathways. These results indicated that this strain has a diverse range of functions in carbohydrate and amino acid metabolism, allowing for more efficient energy conversion. KEGG analysis also revealed numerous metabolic pathways, many of which are predicted to be associated with the biosynthesis of secondary metabolites (Figure S3). A total of 4882 (60.47%) genes were annotated into 24 COG functional categories. These genes were classified into four main categories: cellular, information, metabolism, and poorly. After comparing a protein sequence, it could be assigned to a particular Cluster of Orthologous Groups (COG), and each COG cluster comprised closely related sequences, enabling the sequence’s function to be inferred. The majority of genes evaluated were associated with ‘transcription’, followed by ‘carbohydrate transport and metabolism’, ‘amino acid transport and metabolism’, and ‘signal transduction mechanisms’ (Figure S4).

Carbohydrate-active enzymes (CAZymes) play a role in the complex carbohydrate metabolism. They are understood to be responsible for the synthesis (glycosyl transferases [GT]), degradation (glycoside hydrolases [GHs], polysaccharide lyases [PLs], carbohydrate esterases [CE], and coactive enzymes [AA]), and recognition (carbohydrate-binding modules [CBM]) of all carbohydrates [17]. In the strains of *K. setae*, a total of 441 genes were assigned to the CAZyme family, which consisted of 160 GHs, 10 AAs, 17 CEs, 110 GTs, 3 PLs, and 141 CBMs (Table S3). GHs were found to be the most prevalent CAZymes in the strain. CAZymes are commonly found in all organisms, typically accounting for 1–3% of the gene content. However, they are particularly abundant in microorganisms that degrade plant material, due to the large quantities needed to break down the complex carbohydrates in cell walls [18].

### 2.3. Analysis of Secondary Metabolite Biosynthetic Potential

A total of 40 putative BGCs associated with specialized metabolite production were predicted in the strain’s genome (Table S4). These included four polyketide BGCs (two T1PKS, one T2PKS, and one T3PKS), six butyrolactones BGCs, five non-ribosomal peptide BGCs (NRPS), five terpene clusters, and five lanthipeptides (two lanthipeptide-class-iv, two lanthipeptide-class-i, and one lanthipeptide-class-iii). Additionally, several other BGCs were identified, such as iron chelators, phosphates, aryl polyenes, and thioamide NRPs. BGCs 14, 20, 31, and 35 exhibit 100% similarity with the known BGCs encoding ε-Poly-l-lysine, geosmin, alkylresorcinol, and class IV lanthipeptide, respectively. Two other predicted gene clusters (BGCs 4 and 6) show at least 80% similarity with known compound gene clusters. In addition, there were 34 predicted gene clusters that have less than 25% similarity with known gene clusters. It is worth noting that BGC regions 9, 13, 17, 18, 23, 28, 30, 33, 36, 37, 38, and 39 do not display any similarity with the reference specialized metabolites, indicating the novelty of the BGCs in *Kitasatospora setae*. As a result, the specific biosynthetic products generated by these gene clusters from the *K. setae* strain remain unidentified and necessitate further investigation and exploration.

**Figure 2:**
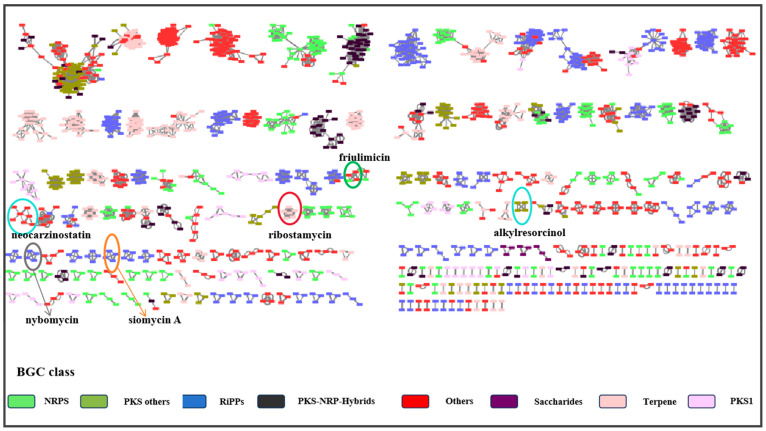
Sequence similarity network of predicted BGCs from the genomes of seventy different Kitasatospora strains, with each node representing a BGC. Different colors represent different categories of BGC, as indicated in the legend. The clusters of genes that partially produce similar biologically active substances are labeled.

Genome-wide data from 69 different previously reported strains of *Kitasatospora* were obtained from the National Center for Biotechnology Information (NCBI) and analyzed using antiSMASH along with the strain identified in this study. The resulting gene cluster data were downloaded and studied. The diversity and novelty of the 2696 putative BGCs from the 70 sets of *Kitasatospora* genomes were assessed using the Biosynthetic Gene Similarity Clustering and Prospecting Engine (BiG-SCAPE) and analyzed by clustering. The sequence similarity network results of the predicted BGCs showed that the 2696 BGCs were classified into eight different categories and organized into 199 gene cluster families (Figure 2).

Based on the Big-SCAPE analysis, it is evident that the gene cluster categories of the 70 strains belonging to the genus *Kitasatospora* are predominantly PKS, NRPS, and RiPPS. The majority of the hypothetical gene clusters observed in this study for *K. setae* exhibit similarities with those of other species within the same genus. However, there are a few gene clusters (e.g., gene clusters 3, 4, 6, and 8) that do not share similarities with gene clusters from other species within the genus.

**Figure 3:**
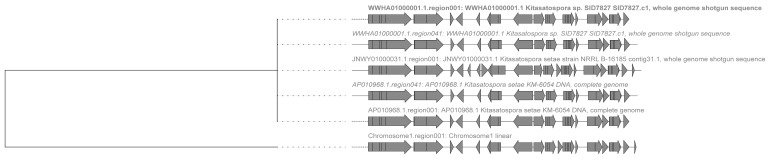
The phylogenetic tree shows six clusters of genes with high similarity within the same genus. The gene cluster sequences in the first five rows belong to *Kitasatospora* sp. SID78276, *Kitasatospora setae* NRRL B-16185, and *Kitasatospora setae* KM-6054. (The strain studied in this paper is in the bottom row).

In the Terpene class, the putative gene cluster 1 of *K. setae* shows similarities with the putative gene clusters of four other strains within the genus *Kitasatospora* (Figure 3). Notably, two of these strains are members of the same species as the strain studied in this research. In addition, two strains (*Kitasatospora* sp. SID78276 and *Kitasatospora setae* KM-6054) each had two putatively similar BGCs. However, the antiSMASH analysis revealed that the predicted gene cluster 1 has a low similarity of 7% to gene clusters known to be responsible for producing ribostamycin. Ribostamycin is an aminoglycoside antibiotic known for its antimicrobial effects against various bacteria such as *E. coli*, *G. aureus*, *Streptococcus*, and *Pneumococcus* [19]. Thus, it is improbable that gene cluster 1, identified by *K. setae*, is involved in the production of a ribostamycin-like metabolite. Therefore, it is not possible to accurately determine the metabolites that may be produced by this gene family. The BiG-SCAPE results indicate that the entire gene cluster consists of a putative peptidoglycan binding domain, a type III restriction enzyme, a helicase conserved C-terminal domain, a KAP family P-loop domain, pentapeptide repeats, a transposase DDF domain, and a putative transposase of IS 4/5 family. The main differences that were observed relate to the deletion of certain ORFs. For instance, the *K. setae* strain studied here lacks two ORFs in front of the core gene PF19086 compared to that of *Kitasatospora setae* NRRL B-16185. Additionally, compared to *Kitasatospora setae* KM-6054, *K. setae* has an extra ORF between the putative peptidoglycan binding domains and putative pentapeptide repeats, and an extra ORF at the end of the gene cluster. Given that multiple gene clusters in this group share identical sequence core motifs and exhibit a significant level of similarity, there is a limited likelihood of novelty within this cluster. It is highly probable that this cluster continues to produce the well-known compound ribostamycin, or its similar derivatives.

**Figure 4:**
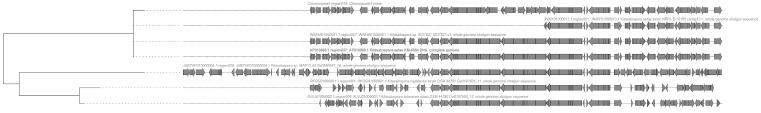
The phylogenetic tree shows seven distinct gene clusters clustered into one gene family. The first row represents the strain examined in this paper, and the following six rows of gene cluster sequences correspond to *Kitasatospora setae* NRRL B-16185, *Kitasatospora* sp. SID78276, *Kitasatospora setae* KM-6054, *Kitasatospora* sp. MAP12-44, and *Kitasatospora niigatensis* DSM 44781.

Within the NRPS category, putative gene cluster 16 of *K. setae* is associated with the putative gene clusters of six other species within the genus, most of which are not the same species as this rare actinomycete (Figure 4). AntiSMASH analysis revealed that gene cluster 16 has the potential to produce analogues of friulimicin, a lipopeptide antibiotic with bacteriostatic activity against a wide range of Gram-positive bacteria such as methicillin-resistant *Enterococci* and *Staphylococcus aureus* [20]. Previous studies have identified 24 open reading frames in the gene cluster, which include several genes used for biosynthesis, as well as four different regulatory genes (*regA*, *regB*, *regC*, and *regD*). Furthermore, it was demonstrated that most friulimicin biosynthesis genes were positively regulated by RegA and RegA, and may be involved in a complex multicomponent regulatory network [21]. The gene cluster 16 reveals several components: core biosynthetic genes (*pstA*, *pstB*, *pstC*, *pstD*), additional biosynthetic genes (*dabA*, *dabB*, *dabC*, *regB*, *lipA*, *lipB*, *lipE*, *mem*, *pip*), transport-related genes (*expA*, *expB*, *mem2*), regulatory genes (*regC*, *regD*, *regE*), and other genes. The main functions of the proteins encoded by biosynthetic genes include condensation, AMP-binding, PP-binding, the synthesis of cysteine, the cleavage of argininosuccinate, etc. Transport genes include the drug-resistance transporter, ABC-2 type transporter, major facilitator transporter, and regulatory genes include the LuxR family DNA-binding response regulator, sensor histidine kinase, and major facilitator transporter. The results of the BiG-SCAPE analysis revealed that gene cluster 16 consists of various genes, including Cytochrome P450, transmembrane secretion effector, the condensation domain, AMP-binding enzyme, and the thioesterase domain. Gene cluster 16 exhibits significant similarities with the gene clusters found in *Kitasatospora* sp. SID7827 and *Kitasatospora setae* KM-6054. However, the gene cluster of *K. setae* has a lesser portion of the phosphopantetheine attachment site compared to the gene clusters of the aforementioned strains. Furthermore, there are notable differences between *K. setae* and *Kitasatospora* niigatensis DSM 44781, as well as *Kitasatospora cineracea* DSM 44780. The primary distinction lies in the transmembrane secretion effector region of the gene cluster, where the genes in front of this region are fundamentally different. Hence, *K. setae* still appears to possess the ability to produce new friulimicin analogues.

In addition to the two gene clusters mentioned above, there are several other gene clusters within the genus that exhibit significant similarities with gene clusters in other species. As previously mentioned, four gene clusters within the genus do not have similar counterparts. Notably, two of these gene clusters have the ability to synthesize bafilomycin and kirromycin, respectively. However, according to the results of the MIBiG, all these gene clusters share similarities with gene clusters found in a specific species within the genus *Streptomyces*.

**Figure 5:**
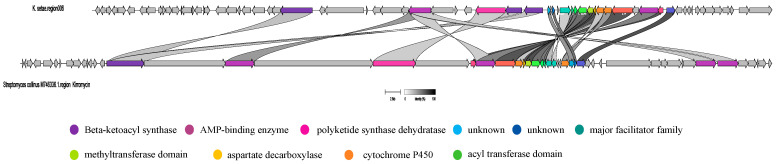
Clinker comparison of gene cluster 6 of *K. setae* with its MIBiG matching resultant gene cluster (kirromycin from *Streptomyces collinus* Tu 365).

**Figure 6:**
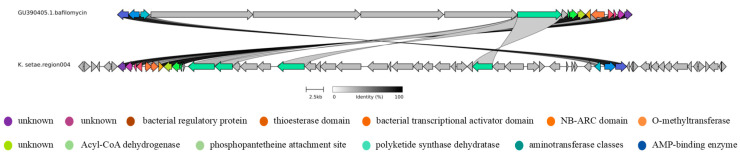
Clinker comparison of gene cluster 4 of *K. setae* with its MIBiG matching resultant gene cluster (bafilomycin from *Streptomyces lohii*).

To study gene clusters 4 and 6 of the strain, we obtained similar gene clusters from NCBI that corresponded to the MIBiG results. These clusters were then imported as GBK files into BigSCAPE for analysis. Notably, the presence of the kirromycin BGC and bafilomycin BGC in *K. setae* was not clustered together with the two BGC sets discovered in the genomes of other species within the same genus, despite their sharing similar core genes. One possible explanation for this discrepancy is that the *K. setae* genome, predicted to have the kirromycin BGC and bafilomycin BGC in antiSMASH, contains a higher number of PKS and NRPS genes compared to other species in the same area. Consequently, we proceeded to analyze the two gene clusters separately using the Clinker tool. The results of this analysis are presented in Figure 5 and Figure 6.

As can be seen in Figure 5 and Figure 6, although there are similarities between the gene cluster of *K. setae* and some of the genes of the MIBiG-matched gene clusters, the biosynthetic gene cluster of *K. setae* shows discontinuities and inconsistencies in the direction of transcription when compared to the relevant BGC of the *Streptomyces* strains.

In Figure 5, both pairs of gene clusters contain genes for Beta-ketoacyl synthase, the phosphopantetheine attachment site, the AMP-binding enzyme, polyketide synthase dehydratase, the acyl transferase domain, the major facilitator family, cytochrome P450, aspartate decarboxylase, and the methyltransferase domain [22]. However, they were transcribed in different directions and were discontinuous overall. Additionally, the gene cluster of the sequenced strains has more genes in the front part, such as the UTRA domain and bacterial regulatory protein, compared to the known gene cluster. The UTRA domain is a conserved ligand-binding structural domain with the main function of DNA binding. It regulates the activity of bacterial transcription factors in response to small molecule binding. Meanwhile, as shown in Figure 6, it is evident that certain parts of this pair of gene clusters exhibit 100% similarity. These include bacterial regulatory protein, the thioesterase domain, O-methyltransferase, and Acyl-CoA dehydrogenase, phosphopantetheine attachment site, and the AMP-binding enzyme, among others. Additionally, there are regions that show some similarities, such as transcriptional regulatory protein, the NB-ARC domain, aminotransferase classes I and II, polyketide synthase dehydratase, and so on. Notably, gene cluster 4 of the sequenced strains was transcribed in the opposite direction compared to the *Streptomyces* gene clusters. Furthermore, certain regions with high similarity were found to be disrupted by other genes. Due to the low similarity between these gene clusters, it is possible that the sequenced strains could produce new kirromycin analogues and bafilomycin analogues.

**Figure 7:**
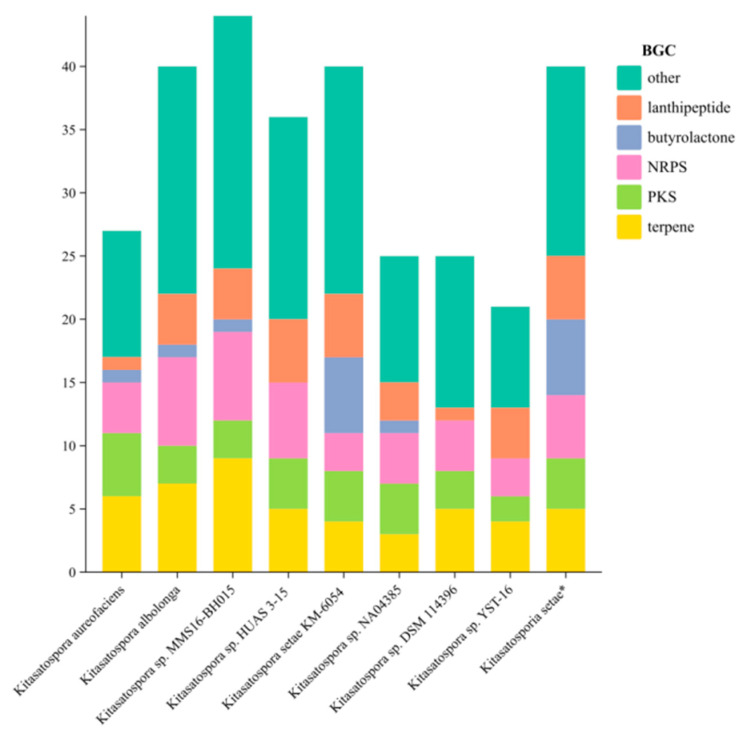
Histogram comparing the number and type of gene clusters between strain *K. setae* and the other eight strains in the genus (the strains studied here have been labelled *).

**Figure 8:**
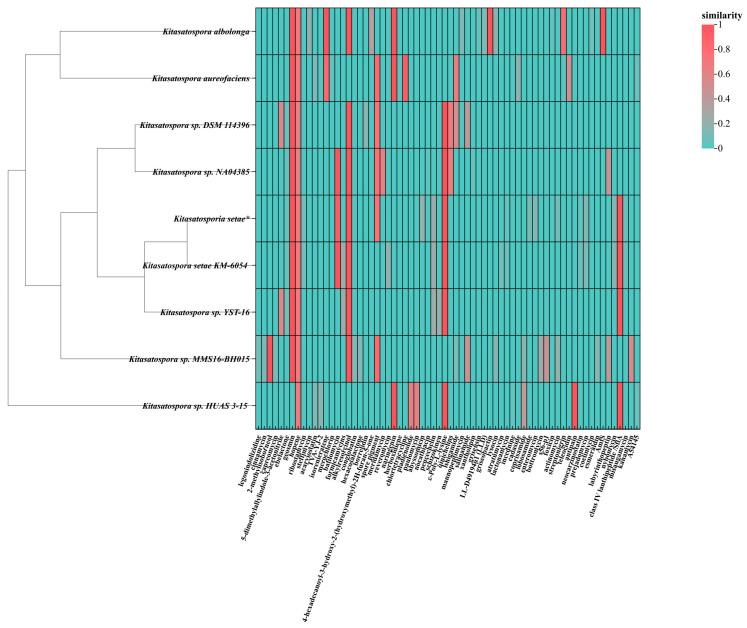
Heat map of predicted products based on gene clusters between strain *K. setae* and eight other strains in the genus (the strains studied here have been labelled *). The horizontal coordinates show the possible secondary metabolites produced by the putative BGC in the nine strains, with the different colors representing the similarity of the BGC products in these strains to the known compounds.

Comparing the sequenced strain *K. setae* with several reported sequences of different species from the genus *Kitasatospora* in the NCBI database showed that the number of biosynthesis gene clusters (BGCs) in strain *K. setae* was significantly higher than some other strains within the genus (Figure 7). Furthermore, the sequenced strain exhibited a greater diversity of BGCs. It is particularly noteworthy that the sequenced strain showed a substantial abundance of gene clusters involved in the synthesis of compounds such as butyrolactones and lanthipeptides compared to other strains. The heat map (Figure 8) illustrated that out of the five main BGC categories—terpene, PKS, NRPS, butyrolactone, and lanthipeptide—most strains of *Kitasatospora* are capable of synthesizing geosmin, hopene, spore pigment, ebelactone, and alkylresorcinol. However, many other secondary metabolites are specific to individual strains. Apart from these five main BGC categories, strain *K. setae* also exhibits several unique BGCs, such as arylpolyene, lanthopeptide, and thioamide-NRP.

Based on the data analyzed for *K. setae*, it was possible to identify more than thirty other gene clusters with very low similarity, indicating the potential for genome-mining. These findings suggest that *K. setae* has the ability to biosynthesize various backbones or classes of natural products.

### 2.4. Molecular Networking Analysis

**Figure 9:**
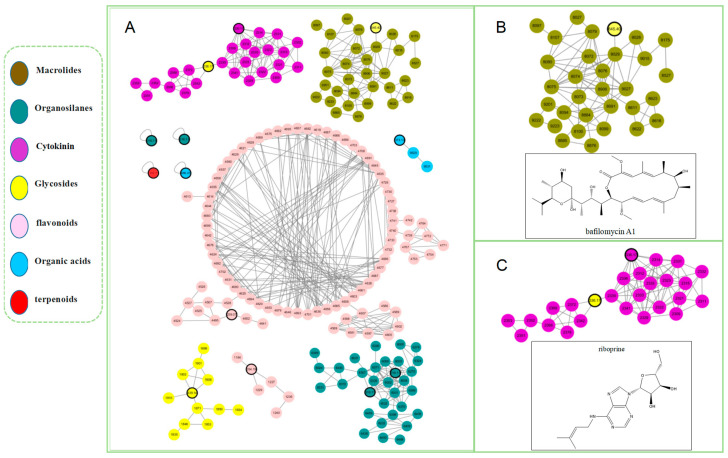
Detected chemistries of the EtOAc extract of *Kitasatospora setae* as generated by HRESIMS, which were analyzed via the GNPS platform. (**A**) Structural annotations for molecular families, wherein the color of the nodes denotes the structural annotation at the superclass level. (**B**) Observations of molecular family A allow for the tentative identification of bafilomycin A and many related analogues. (**C**) Observations of molecular family B allow for the tentative identification of Riboprine and many related analogues.

**Figure 10:**
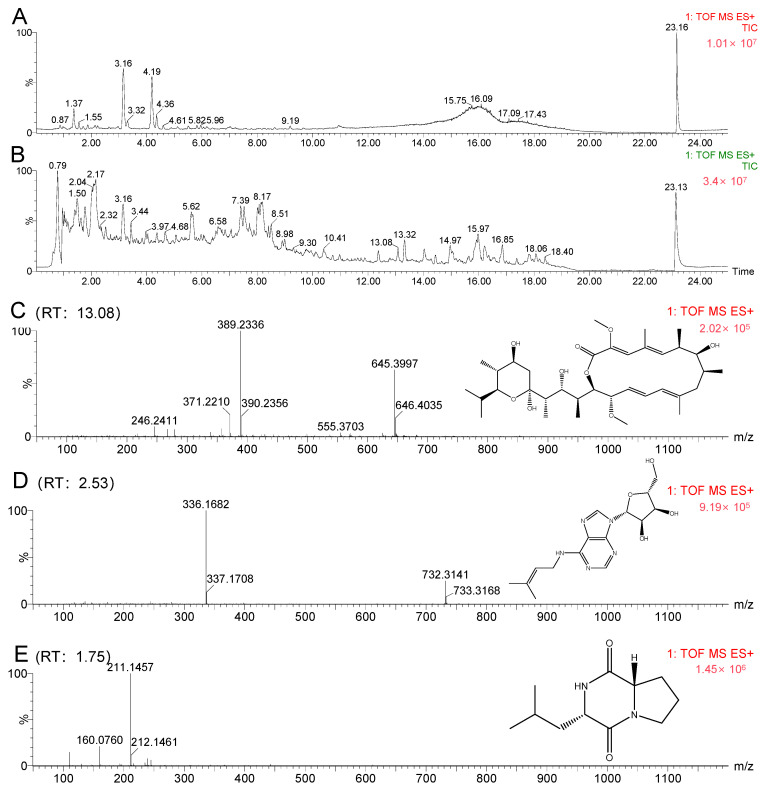
(**A**) The chromatogram of the blank control (pure fermentation medium). (**B**) The chromatogram of the crude extract of *K. setae*. (**C**) Combining GNPS prediction with Cytoscape visualization confirmed that the compound eluting at 13 min in the chromatogram is bafilomycin A1. (**D**) Likewise, the compound eluting at 2.5 min was identified as lipoadenosine. (**E**) The compound at 1.8 min was confirmed to be cyclo(proline-leucine).

A molecular networking analysis of the *K. setae* strain revealed the presence of specific alkaloids, glycosides, and macrolides in the organic extract. Upon manual examination of these annotations, two intriguing compounds, bafilomycin A and riboprine, were identified in molecular families A and B, respectively (Figure 9). Further analysis of the main constituents of the mixture, using MS and collision-induced MS/MS (MS^2^), showed that certain metabolites in the organic extract produced a product ion with *m*/*z* 645.3997 [M + Na]^+^, corresponding to the formula C_35_H_58_O_9_ (calculated value 622.4080). Evaluating both the Global Natural Products Social Molecular Networking (GNPS) analysis results and the HRESIMS chromatogram, it was determined that the peak at around 13 min had the same mass as the molecule of interest (*m*/*z* 645.3997) (Figure 10C). There was also a clear difference between the chromatograms of the blank control (pure fermentation medium) and the strain *K. setae* in Figure 10. The retention times of the predicted compounds were determined by analyzing the molecular network visualized in Cytoscape. Peaks with corresponding retention times were then identified on the chromatograms of the crude extracts. Compounds with a retention time of 13 min were confirmed to be bafilomycin A by observing the mass spectra. This finding demonstrated the potential of the rare actinomycete *K. setae* to produce a wide range of bafilomycin analogues under existing laboratory conditions. Bafilomycin A is a macrolide antibiotic that reversibly inhibits terminal autophagy and has inducible regulatory effects on cell proliferation. This result was also consistent with the product of gene cluster 4, as analyzed by antiSMASH in the previous section. The combined use of genome mining and molecular networks enables the more accurate and efficient identification of metabolites such as bafilomycins.

By utilizing the GNPS tool, we hypothesized the possible prespostulated potential existence of N6-isopentenyladenosine (iPA) in the crude extract (Figure 10D), as indicated by a product ion peak at *m*/*z* 336.167 [M + H]^+^ with the molecular formula C_15_H_21_N_5_O_4_ (calculated value 335.3600). The retention times of the predicted compounds were determined by analyzing the molecular network visualized in Cytoscape. Peaks with corresponding retention times were then identified on the chromatograms of the crude extracts. Compounds with a retention time of 2.5 min were confirmed to be iPA by observing the mass spectra. IPA is a cytokinin, and previous studies have reported the antitumor action of iPA in various types of tumors, such as breast cancer cells, bladder carcinoma, colon cancer cells, melanoma cells, and others [23,24,25,26]. Furthermore, iPA exhibited pleiotropic properties that can interfere with tumor growth at multiple levels. It acted as an angiogenesis inhibitor and an immunomodulatory agent, selectively activating natural killer cells.

The findings from GNPS analyses indicated that the extract potentially contains flavonoids, which exhibit binding abilities to biological macromolecules and a potent antioxidant impact on cell membranes. These results hold promise for potential therapeutic applications [27]. However, it is important to note that this class of compounds is primarily found in plants and is unlikely to be produced by microorganisms, which may affect the accuracy of the results obtained from GNPS analysis. In addition, we speculate that the crude extract might contain cyclic dipeptides (e.g., proline-leucine) (Figure 10E) with antimicrobial activity [28].

However, it is noteworthy that the majority of nodes in the molecular networking did not correspond to known molecules with MS/MS spectra in the database. This highlighted the significant potential of *K. setae* for generating structurally interesting bioactive secondary metabolites. Interestingly, GNPS-predicted compounds differed significantly from those predicted by antiSMASH genomic analysis, which may be attributed to certain BGCs being misannotated or silenced under current laboratory conditions.

Purification of the organic extract yielded the isolation of compound **1** as a pale yellow powder. An analysis of the ^1^H and ^13^C NMR spectroscopic data supported the idea that the compound was indeed bafilomycin A. The ^1^H and ^13^C NMR spectra of compound **1** can be found in Figures S5 and S6, HR-ESI(+) MS *m*/*z* 622.84. In addition, we identified three other compounds from the crude extract. Compound **2** (1-Acetyl-β-carboline) was a white powder with ^1^H and ^13^C NMR (see Figures S7 and S8), and HR-ESI(+) MS *m*/*z* 210.08. Compound **3** (methyl 4-Hydroxy-3-methoxyacetophenone) was a white powder with ^1^H and ^13^C NMR (see Figures S9 and S10), and HR-ESI(+) MS *m*/*z* 182.06. Compound **4** (turnagainolide) was a white powder, with ^1^H and ^13^C NMR (see Figures S11 and S12), and HR-ESI(+) MS *m*/*z* 556.33. It is worth noting that many of the compounds identified by the GNPS analysis were not obtained in pure form from the crude extracts; this may be due to their lower abundance or losses during the experimental process.

## 3. Discussion

In this study, the metabolic potential of the rare actinomycete *K. setae* was investigated using a combined genomic–metabolomic approach. The analysis of nanopore sequencing results revealed a high GC content in the base sequence of this strain, posing challenges for primer design and amplification during the later stages of assay development. Additionally, studying prophage sequences not only facilitates antibiotic resistance acquisition but also aids in identifying specific antibiotics.

An analysis of antiSMASH revealed a total of 40 putative BGCs associated with secondary metabolite production in the genome of this strain. These BGCs encompassed polyketides, butyrolactones, non-ribosomal peptides (NRPS), terpene clusters, lanthipeptides, iron carriers, phosphates, aryl polyenes, and thioamide NRPs. Among these, only six BGCs exhibited >80% similarity to known BGCs, while the remaining BGCs displayed low similarity, suggesting that the BGCs of *K. setae* were novel. Further investigation and exploration are necessary to understand the unique metabolites produced by these BGCs and their potential significance.

By comparing the BGCs of the sequenced strains with those of other strains that are in the same genus but different species in the NCBI database, the results from Clinker and Big-SCAPE also highlighted the potential of the sequenced strains to produce a diverse range of novel compounds.

Gene cluster 35 exhibited complete similarity to class IV lanthipeptide/SflA, which represents a minor fraction of the over 100 identified lanthipeptides reported so far. Lanthipeptides exerted antibacterial activity primarily by binding to lipid II, which is not antagonized by vancomycin [29]. Therefore, this class of compounds provides a promising alternative for the treatment of drug-resistant bacterial infections [30]. The product of gene cluster 31 was predicted to be completely homologous to alkylresorcinol. Alkylresorcinols, which have recently been discovered in cereals, have been shown to inhibit enzyme activity, prevent bacterial or fungal infections, reduce cholesterol absorption, and prevent cancer [31].

Gnee cluster 20 was suggested to produce geosmin. Geosmin is a volatile substance with an earthy odor that is produced by various microorganisms during their metabolism [32]. Due to its extremely low odor threshold, this compound is a significant odor pollutant in drinking water [33]. The predicted product of gene cluster 14 was ε-Poly-L-lysine, a cationic polymeric polypeptide. ε-Poly-L-lysine exhibits a broad antimicrobial spectrum with the significant inhibition and killing of yeasts, Gram-positive bacteria and Gram-negative bacteria. By combining ε-PL with other food additives, preservative activity can be greatly enhanced [34,35]. The product of gene cluster 6 was suggested to be 84% similar to kirromycin. Kirromycin was a bacterial protein synthesis inhibitor produced by *Streptomyces*, and it was assembled from a hybrid modular polyketide synthase (PKS)/non-ribosomal peptide synthase (NRPS) [36]. Gene cluster 4 shared 94% similarity with that reported for bafilomycin, a macrolide antibiotic that was initially discovered in *Streptomyces* sp. This antibiotic is known to inhibit Gram-positive bacteria and fungi, and it acts as an inhibitor of K^+^-dependent ATPase in *E. coli*. Furthermore, bafilomycin exhibits antibacterial, anti-fungal, antitumor, anti-osteoporotic, and immunosuppressive properties [37,38]. However, the synthesis of analogues is limited due to the structural complexity of these compounds.

Upon comparison with other strains of the genus *Kitasatospora* in the NCBI database, it was observed that strain *K. setae* possesses a substantial number of gene clusters involved in the synthesis of butyrolactones and anthipeptides, among others. Furthermore, aside from these five major classes of BGCs, strain *K. setae* exhibits several unique BGCs. In addition, the data were analyzed using genomic functional annotation methods. The functional categories were determined using different annotation methods, and the major metabolic pathways were identified. Overall, the data provided a rough understanding of the functional categories and major metabolic pathways.

The results of the GNPS analysis demonstrated that the secondary metabolites of the strain included compounds such as alkaloids and macrolides. Upon manual examination of these annotations, it was found that the primary compounds identified were bafilomycin, riboprine, flavonoids, and arnicolide D. These compounds possess antimicrobial, antioxidant, and antitumor activities. Comparing the genome analysis results with the GNPS findings, it was observed that some genes were in a silent state and did not produce the corresponding metabolites. The known bafilomycin was obtained by purification using reverse-phase MPLC and HPLC. To activate these silent genes, strategies like OSMAC can be employed.

## 4. Materials and Methods

### 4.1. Instruments Used in the Experiment

All the instrumentation used in this experiment included a double single-side purification workbench (Shanghai Shuo Guang Electronic Technology Corporation, Shanghai, China), Intelligent Biochemical Incubator (Shanghai Shuo Guang Electronic Technology Corporation, Shanghai, China), autoclave (Panasonic Healthcare Corporation, Tokyo, Japan), high-speed centrifuge (Eppendorf, Hamburg, Germany), Large-Capacity Combined Shock Incubator (Shanghai Minquan Instrument Corporation, Shanghai, China), FLEXA Purification System (Tianjin, China, Agela Technologies), Rotary Evaporator (Ningbo Hangjing Biotechnology Corporation, Ningbo, China), Waters Xevo G2-XS Q-Tof mass spectrometer (Waters Corporation, Milford, MA, USA), Agilent HPLC 1260 Infinity instrument (Agilent Technologies Inc., Santa Clara, CA, USA), and 600 MHz Bruker AVANCE NEO spectrometer (Bruker, Karlsruhe, Germany).

### 4.2. Rare Actinomycete Strains

The strain *Kitasatospora setae* was derived from the soil of Setagaya, Tokyo, Japan.

### 4.3. Genomic DNA Preparation and Purification

The strain was incubated in YMG medium at 28 °C for 3 days with shaking at 200 rpm, while the cultured organisms were extracted by centrifugation. YMG medium was formulated as follows: 20 g of soluble starch, 10 g of glucose, 5 g of yeast extract, 5 g of malt extract, and 0.5 g of calcium carbonate per 1 L of distilled water.

The desired amount of tissue samples was placed into a grinder and processed into powder. The powder was scraped into a 5 mL centrifuge tube, to which 3.5 mL of Microbial Lysis Buffer 1 was added, along with 100 µL of Proteinase K and 30 µL of RNaseA, followed by upside-down mixing multiple times. After incubating at 37 °C for 30 min, 1.2 mL of Microbial Lysis Buffer 2 was added, then upside-down mixing commenced. The reaction was incubated at 55 °C for 120 min. This was followed by column-based genomic DNA purification and elution of the DNA precipitate.

### 4.4. High-Throughput Sequencing

Nanopore sequencing, a so-called third-generation approach, was used to evaluate the genome of *K. setae*. The library preparation method was straightforward: DNA ends were repaired and end-prepped/dA-tailed, and then sequencing adapters were ligated onto the prepared ends. The six steps in the process were as follows: sample QC, DNA fragment selection, end prep and nick repair, ligation of sequencing adapters, validation of the library, and loading and sequencing.

### 4.5. Assembly and Genome Annotation

Clean data were assembled using SPAdes assembly software (v3.9.0), and the optimal assembly results of each sample were obtained after multiple adjustments. Then, reads were aligned to the assembled contig and the assembled results were optimized through the overlap of the pair end of the reads and contig.

The function annotation was accomplished by an analysis of protein sequences. Gene annotation was used for the functional analysis of all protein-coding genes, including predictions of information such as motifs, domains, protein functions, and metabolic pathways. Genes were aligned with databases to obtain their corresponding annotations. To ensure the biological meaning was correct, the highest-quality alignment results were chosen as gene annotations. Function annotations were completed by blasting genes with different databases using Diamond software (v2.1.8), such as GO, COG, KEGG, and CAZy.

### 4.6. Prediction of Secondary Metabolite Gene Clusters

The genomic data obtained from the sequencing were uploaded to the antiSMASH website for analysis to predict the secondary metabolic potential of the *K. setae* strain. The antiSMASH 7.0 [39] website and MIBIG were used to analyze and categorize the gene cluster data. The BiG-SCAPE [40] algorithm, also known as the Biosynthetic Gene Similarity Clustering and Prospecting Engine, was employed to generate sequence similarity networks, which were then visualized using Cytoscape (v3.9.1). Clinker was used to compare clusters of genes that exhibited sequence similarity in the BiG-SCAPE results.

Genome-wide data from 69 different previously reported strains of *Kitasatospora* were obtained from NCBI and analyzed using antiSMASH, along with the strain described here. The diversity and novelty of the 2696 putative BGCs from the 70 *Kitasatospora* genomes were assessed using BiG-SCAPE and analyzed by clustering. The tools and methods used for the specific genomic analyses conducted here partially followed the reported methods that were previously used to study the secondary metabolic potential of *Kutzneria* [41].

### 4.7. Chemical Analysis of Secondary Metabolites

Colonies of the strain *K. setae* growing on a Petri dish containing ISP2 solid medium were inoculated into 1000 mL flasks containing 300 mL of YMG liquid medium, then incubated for 4 days at 28 °C with rotary shaking (200 rpm) to obtain a seed culture. Subsequently, a large-scale fermentation was performed in 100 × 1000 mL flasks, with each flask equally inoculated with 10% seed solution, then cultured for 12 days at 28 °C under static conditions, and finally extracted with ethyl acetate (1:1, *v*/*v*) three times. The organic phase was concentrated via rotatory evaporation under vacuum to yield 10.3 g of an organic extract.

The EtOAc extract underwent column chromatography on silica gel with a gradient elution of EtOAc/PE (0:100–100:0, *v*/*v*), resulting in the separation of 7 fractions (Fr.1–Fr.7). Medium-pressure liquid chromatography (MPLC) was performed on a FLEXA Purification System using a 15 µm ODS column (Santai Technologies, Inc., Changzhou, China). Fr.4 was subjected to further fractionation via reverse-phase MPLC using a gradient elution of 20–100% MeOH/H_2_O, leading to the isolation of 4 subfractions (Fr.4.1–Fr.4.4). Subfraction Fr.4.3 was subsequently purified through semi-preparative HPLC, employing a YMC-Triart C18 column with an isocratic elution of 35% ACN/ H_2_O, yielding compound **1** (2.1 mg, *t*_R_ = 34 min). Subfraction Fr.4.2 underwent a similar purification process using the same column with an isocratic elution of 20% ACN/ H_2_O, resulting in the isolation of compound **2** (1.7 mg, *t*_R_ = 21 min). Fr.2 was also further fractionated by reverse-phase MPLC with a gradient elution of 15–100% MeOH/H_2_O, resulting in the generation of three subfractions (Fr.2.1–Fr.2.3). Subfraction Fr.2.3 was then purified using semi-preparative HPLC with a YMC-Triart C18 column and an isocratic elution of 17% ACN/H_2_O, leading to the isolation of compound **3** (2.2 mg, *t*_R_ = 26 min). Fr.6 underwent a similar process of further fractionation by reverse-phase MPLC with a gradient elution of 50–100% MeOH/H_2_O, resulting in the formation of five subfractions (Fr.6.1–Fr.6.5). Subfraction Fr.6.4 was subsequently purified through semi-preparative HPLC using a YMC-Triart C18 column with an isocratic elution of 75% ACN/H_2_O, leading to the isolation of compound **4** (2.4 mg, *t*_R_ = 43 min).

Compound **1** was a pale yellow powder: ^1^H and ^13^C NMR (see Figures S5 and S6); HR-ESI(+) MS *m*/*z* 622.4081. Compound **2** was a white powder: ^1^H and ^13^C NMR (see Figures S7 and S8); HR-ESI(+) MS *m*/*z* 210.0793. Compound **3** was a white powder: ^1^H and ^13^C NMR (sees Figures S9 and S10); HR-ESI(+) MS *m*/*z* 182.0579. Compound **4** was a white powder: ^1^H and ^13^C NMR (see Figures S11 and S12); HR-ESI(+) MS *m*/*z* 556.3261.

To investigate the specialized metabolites biosynthesized by *K. setae*, the organic extract was dissolved in MeOH and analyzed using High-Resolution Electrospray Ionization Mass Spectrometry (HRESIMS). The analytical tool Global Natural Products Social Molecular Networking (GNPS) was employed to identify the characteristic molecular networks of metabolites based on their MS/MS spectrav [42]. Feature-based molecular networking (FBMN) was utilized as a powerful processing method to visualize and annotate the complex untargeted HRESIMS metabolite profiling analyses of natural extracts. The resulting FBMN was then visualized using Cytoscape after data generation and conversion.

## 5. Conclusions

In this study, we obtained a new, high-quality, rare actinomycete genome, designated as *K. setae*, through whole-genome sequencing and assembly. Analysis of the genome revealed the presence of several secondary metabolic enzymes encoded by this strain. Notably, the antiSMASH results indicated that strain *K. setae* harbored a total of 40 secondary metabolite BGCs, including four polyketide synthases (PKSs), five nonribosomal peptide synthetases (NRPSs), six butyrolactones, five terpenes, and five lanthipeptides. Intriguingly, 12 of these BGCs were unidentified and possessed the potential to produce novel secondary metabolites. Furthermore, the antiSMASH analysis predicted that strain *K. setae* has the ability to produce antibiotics such as bafilomycin and kirromycin. Meanwhile, the analysis based on LC-MS and GNPS molecular networking indicated that this strain has the potential to produce secondary metabolites (SM) with biological activity. This strain could produce bafilomycin analogues with improved bioactivity. However, upon combining the genome with GNPS, it was observed that most of the gene clusters responsible for SM production were inactive. Therefore, future research should prioritize the activation of these dormant gene clusters in the strain to enhance the production of secondary metabolites. This can be achieved through various approaches, such as the OSMAC strategy, incubation with activator molecules, and heterologous gene expression.

## Data Availability

The complete genome sequence of *Kitasatospora setae* was uploaded to BioProject and Sequence Read Archive (SRA) under the accession number PRJNA997087.

## Supplementary Materials

The following supporting information can be downloaded at: https://www.mdpi.com/article/10.3390/antibiotics13050459/s1, Figure S1: Gene length distribution map, Figure S2: GO Functional Annotation Distribution Chart, Figure S3: KEGG Functional Annotation Distribution Chart, Figure S4: COG Functional Annotation Distribution Chart, Figure S5: ^13^C NMR (600 MHz, CDCI_3_) spectrum of bafilomycin, Figure S6: ^1^H NMR (600 MHz, CDCl_3_) spectrum of bafilomycin, Figure S7: ^13^C NMR (600 MHz, CDCI_3_) spectrum of 1-Acetyl-β-carboline, Figure S8: ^1^H NMR (600 MHz, CDCl_3_) spectrum of 1-Acetyl-β-carboline, Figure S9: ^13^C NMR (600 MHz, DMSO) spectrum of methyl 4-Hydroxy-3-methoxyacetophenone, Figure S10: ^1^H NMR (600 MHz, DMSO) spectrum of methyl 4-Hydroxy-3-methoxyacetophenone, Figure S11: ^13^C NMR (600 MHz, DMSO) spectrum of turnagainolide, Figure S12: ^1^H NMR (600 MHz, DMSO) spectrum of turnagainolide; Table S1: General genomic features of the strain *K. setae*, Table S2: Non-coding RNA statistics of the strain *K. setae*, Table S3: Annotated statistical table of carbohydrase classification, Table S4: Summary of antiSMASH analysis results of sequenced strains.
