# Supplementary material for: Genomic Analysis of Kitasatospora setae to Explore Its Biosynthetic Potential Regarding Secondary Metabolites
Source: Antibiotics (Basel). 2024 May 16;13(5):459. doi: 10.3390/antibiotics13050459 (PMC11117518; doi:10.3390/antibiotics13050459)

# Supplementary Materials

**Genomic Analysis of *Kitasatospora setae* to Explore Its Biosynthetic Potential Regarding Secondary Metabolites**

**Yutong Xue,[1] Zhiyan Zhou,[2] Fangjian Feng,[1] Hang Zhao,[1] Shuangling Tan,[1] Jinling Li,[1] Sitong Wu,[3] Zhiran Ju,[3] Shan He,* [1, 2] and Lijian Ding*[1, 2]**

[1]Li Dak Sum Yip Yio Chin Kenneth Li Marine Biopharmaceutical Research Center, Ningbo University, Ningbo, 315211 China;
[2]School of Pharmacy, Ningbo University, Ningbo, 315211 China;
[3]School of Pharmaceutical Sciences, Zhejiang University of Technology, Hangzhou 310014, China.

*Corresponding Authors： heshan@nbu.edu.cn (S.H) ;dinglijian@nbu.edu.cn (L.D)

# Table of contents

Table S1. General genomic features of the strain *K. setae*...............................................................3

Table S2. Non-coding RNA statistics of the strain *K. setae*............................................................3

Table S3. Annotated statistical table of carbohydrase classification...............................................3

Table S4. Summary of antiSMASH analysis results of sequenced strains......................................4

Figure S1. Gene length distribution map .......................................................................................5

Figure S2. GO Functional Annotation Distribution Chart ..............................................................6

Figure S3. KEGG Functional Annotation Distribution Chart..........................................................7

Figure S4. COG Functional Annotation Distribution Chart.............................................................8

Figure S5. $^{13}C$ NMR (600 MHz, $CDCI_3$) spectrum of bafilomycin.................................................9

Figure S6. $^{1}H$ NMR (600 MHz, $CDCl_3$) spectrum of bafilomycin.................................................10

Figure S7. $^{13}C$ NMR (600 MHz, $CDCI_3$) spectrum of 1-Acetyl-β-carboline..................................11

Figure S8. $^{1}H$ NMR (600 MHz, $CDCl_3$) spectrum of 1-Acetyl-β-carboline...................................12

Figure S9. $^{13}C$ NMR (600 MHz, DMSO) spectrum of methyl 4-Hydroxy-3-methoxyacetoph enone..........................................................................................................................................13

Figure S10. $^{1}H$ NMR (600 MHz, DMSO) spectrum of methyl 4-Hydroxy-3-methoxyacetoph enone..........................................................................................................................................14

Figure S11. $^{13}C$ NMR (600 MHz, DMSO) spectrum of turnagainolid e...............................................................................................................................................15

Figure S12. $^{1}H$ NMR (600 MHz, DMSO) spectrum of turnagainolid e...............................................................................................................................................16

Table S1. General genomic features of the strain *K. setae*

| Item | Value |
| --- | --- |
| Total length of genome | 7,552,416 bp |
| Genome Size | 8,686,114 bp |
| GC Content | 74.42% |
| Total Number(Number of genes) | 8,073 |
| Average Length | 935.52 bp |

Table S2. Non-coding RNA statistics of the strain *K. setae*

| Type | Copy Number | Average Length | Total Length | % in Genome |
| --- | --- | --- | --- | --- |
| tRNA | 72 | 76.52 | 5510 | 0.0634 |
| 5s_rRNA | 9 | 115.33 | 1038 | 0.0119 |
| 16s_rRNA | 9 | 1510.22 | 13592 | 0.1546 |
| 23s_rRNA | 9 | 3099.88 | 27899 | 0.3211 |
| sRNA | 65 | 61.95 | 4027 | 0.0464 |

Table S3. Annotated statistical table of carbohydrase classification

| Sample Name | AAs Number | CBMs Number | CEs Number | GHs Number | GTs Number | PLs Number |
| --- | --- | --- | --- | --- | --- | --- |
| *K.setae* | 10 | 141 | 17 | 160 | 110 | 3 |

## Table S4. Summary of antiSMASH analysis results of sequenced strains

| Cluster | Gene Cluster type | from | to | Most similar known cluster (MIBIG) | Similarity | MIBIG BGC-ID |
|---|---|---|---|---|---|---|
| 1 | terpene | 22,957 | 42,676 | ribostamycin | 7% | AJ748131.1 |
| 2 | butyrolactone | 122,007 | 129,948 | neocarzinostatin | 6% | AY117439.1 |
| 3 | NRPS | 139,093 | 186,193 | actinomycin D | 10% | HM038106.1 |
| 4 | T1PKS,transAT-PKS | 368,394 | 461,480 | bafilomycin B1 | 94% | GU390405.1 |
| 5 | T2PKS | 507,096 | 579,539 | spore pigment | 75% | AB070937.1 |
| 6 | transAT-PKS,NRPS | 726,891 | 836,710 | kirromycin | 84% | AM746336.1 |
| 7 | terpene | 837,953 | 858,757 | ebelactone | 5% | LT608336.1 |
| 8 | NRPS-like | 1,156,705 | 1,196,856 | lobosamide | 10% | KT209587.1 |
| 9 | butyrolactone | 1,299,227 | 1,309,074 | | | |
| 10 | thioamide-NRP | 1,315,259 | 1,369,573 | enteromycin | 12% | MW367897.1 |
| 11 | T1PKS | 1,378,574 | 1,432,537 | abyssomicin | 21% | KY432814.1 |
| 12 | arylpolyene | 1,443,682 | 1,484,071 | lobosamide | 10% | KT209587.1 |
| 13 | lanthipeptide class III | 1,694,680 | 1,716,776 | | | |
| 14 | NAPAA | 1,885,602 | 1,919,420 | ε-Poly-L-lysine | 100% | LC517046.1 |
| 15 | lanthipeptide-class-i | 2,173,931 | 2,198,143 | nybomycin | 14% | MH924838.1 |
| 16 | NRPS,butyrolactone | 2,232,774 | 2,291,769 | friulimicin A/friulimicin B | 15% | AJ488769.3 |
| 17 | RiPP-like | 2,592,231 | 2,602,101 | | | |
| 18 | NI-siderophore | 2,773,325 | 2,786,885 | | | |
| 19 | lassopeptide | 3,558,043 | 3,578,916 | siomycin A | 7% | FJ436355.1 |
| 20 | terpene | 3,671,554 | 3,692,669 | geosmin | 100% | AL645882.2 |
| 21 | LAP,lanthipeptide-class-ii | 3,713,263 | 3,739,509 | goadsporin | 12% | AB205012.1 |
| 22 | butyrolactone | 3,841,539 | 3,850,078 | prejadomycin/rabelomycin | 14% | AY034378.2 |
| 23 | lassopeptide | 4,169,815 | 4,207,518 | | | |
| 24 | indole | 4,556,293 | 4,577,191 | 5-isoprenylindole-3-carboxylate β-D-glycosyl ester | 23% | KT895008.1 |
| 25 | NRPS-like | 5,013,569 | 5,055,401 | lactonamycin | 5% | EU147298.1 |
| 26 | LAP,PKS-like,T1PKS,other | 5,678,906 | 5,748,328 | tambjamine BE-18591 | 21% | NZ_JODZ01000004.1 |
| 27 | lanthipeptide-class-iv | 6,088,254 | 6,111,360 | misaugamycin A/misaugamycin B | 5% | QHKJ02000004.1 |
| 28 | butyrolactone | 6,219,340 | 6,228,380 | | | |
| 29 | phosphonate | 6,245,172 | 6,268,662 | phosphonoacetic Acid | 23% | NZ_JNXE01000019.1 |
| 30 | RiPP-like | 6,635,929 | 6,643,235 | | | |
| 31 | T3PKS | 6,744,344 | 6,785,381 | alkylresorcinol | 100% | AP009493.1 |
| 32 | terpene | 6,800,404 | 6,825,170 | hopene | 69% | AL645882.2 |
| 33 | terpene | 7,323,232 | 7,344,428 | | | |
| 34 | NI-siderophore | 7,355,304 | 7,369,954 | peucechelin | 25% | CP022438.1 |
| 35 | lanthipeptide-class-iv | 7,571,216 | 7,593,840 | class IV lanthipeptide/SflA | 100% | NZ_JOIU01000038.1 |

| | | | | | | |
|---|---|---|---|---|---|---|
| 36 | other | 7,748,543 | 7,789,523 | | | |
| 37 | butyrolactone | 7,820,293 | 7,829,716 | | | |
| 38 | lanthipeptide-class-i | 8,208,697 | 8,232,418 | | | |
| 39 | RiPP-like | 8,472,593 | 8,484,470 | | | |
| 40 | butyrolactone | 8,636,036 | 8,647,619 | neocarzinostatin | 4% | AY117439.1 |

Figure S1. Gene length distribution map (The horizontal coordinate is the gene length and the vertical coordinate is the number of genes corresponding to the gene length.)

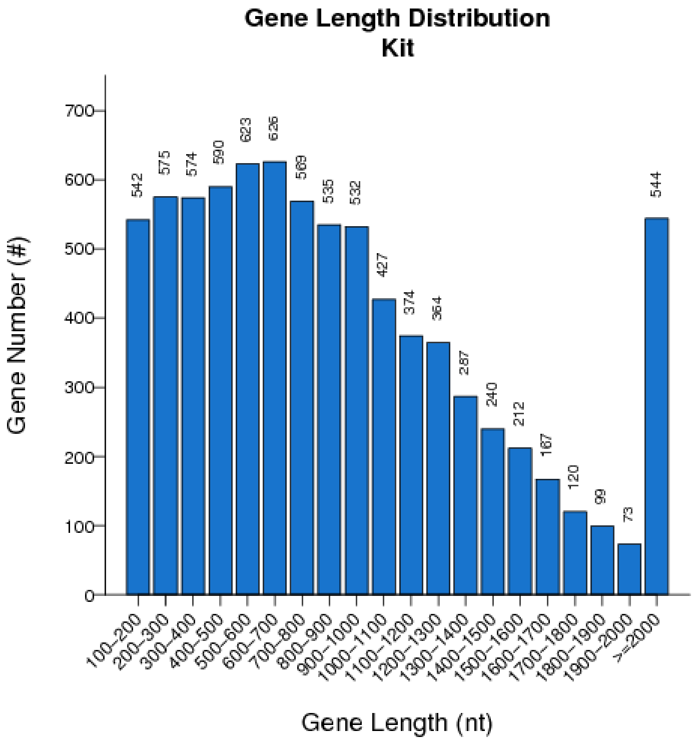

Figure S2. GO Functional Annotation Distribution Chart (The vertical coordinate is the annotation entry and the horizontal coordinate is the number of genes corresponding to the entry). Three main categories are contained (Cellular Component, Molecular Function, Biological Process).

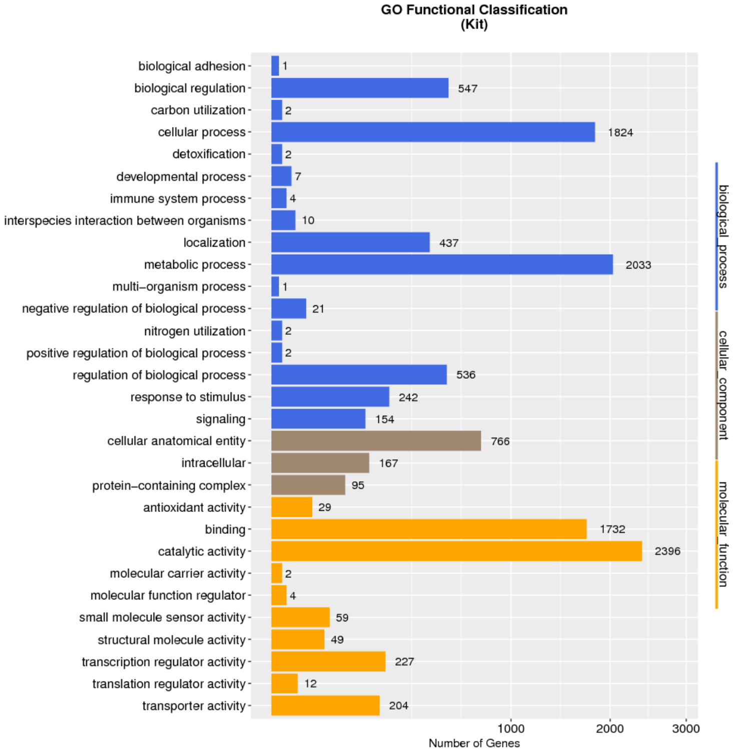

Figure S3. KEGG Functional Annotation Distribution Chart (The vertical coordinate is the annotation entry and the horizontal coordinate is the number of genes corresponding to the entry), This is divided into six categories: cellular processes, environment, genetics, human diseases, metabolism, and organism systems.

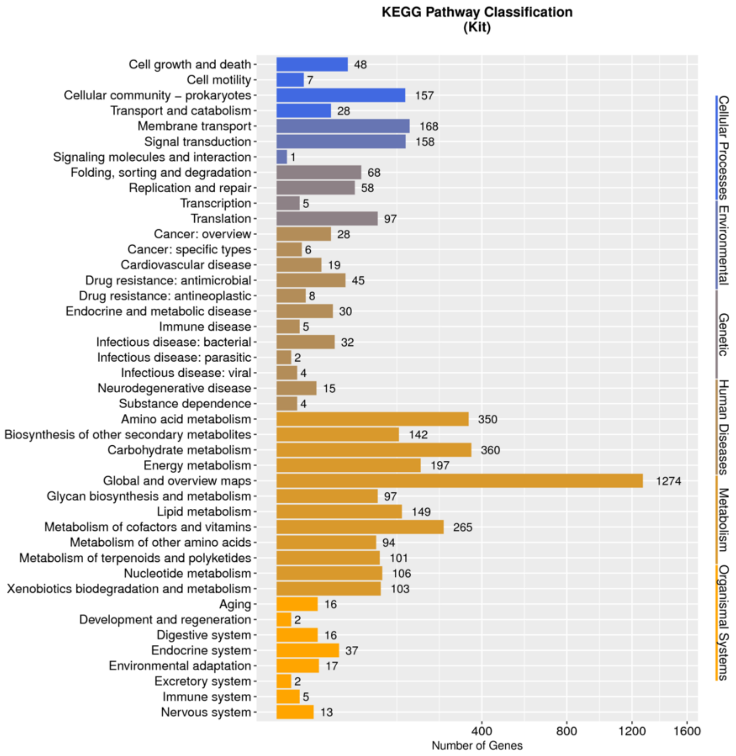

Figure S4. COG Functional Annotation Distribution Chart (The vertical coordinate is the annotation entry and the horizontal coordinate is the number of genes corresponding to the entry), Genes were classified into four main categories: cellular, information, metabolism, and poorly.

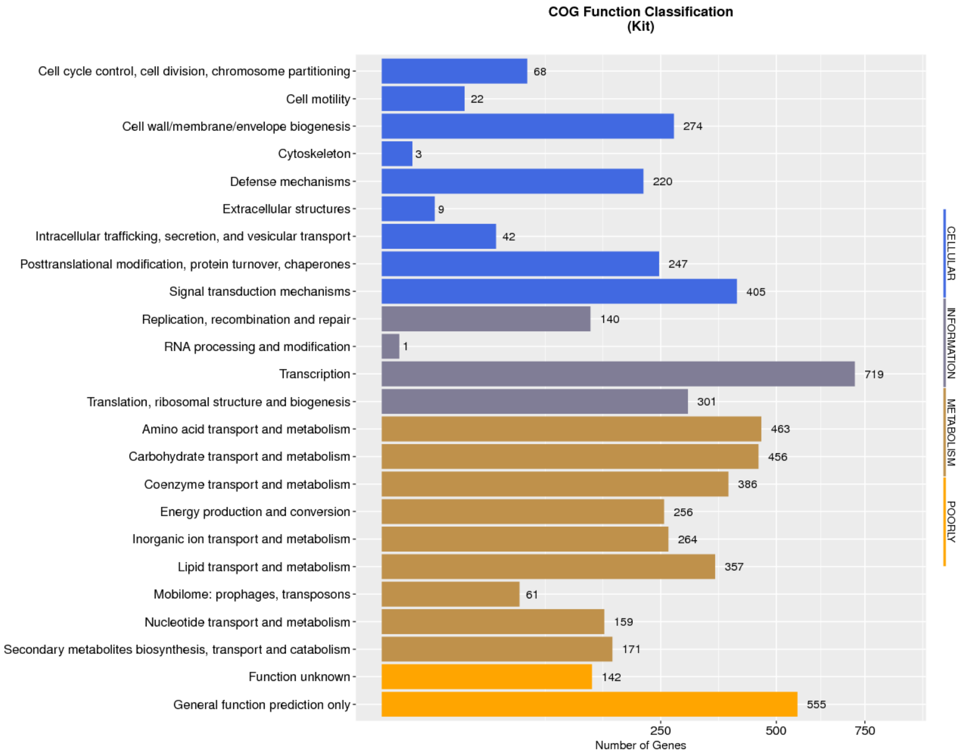

Figure S5. $^{13}C$ NMR (600 MHz, $CDCl_3$) spectrum of bafilomycin.

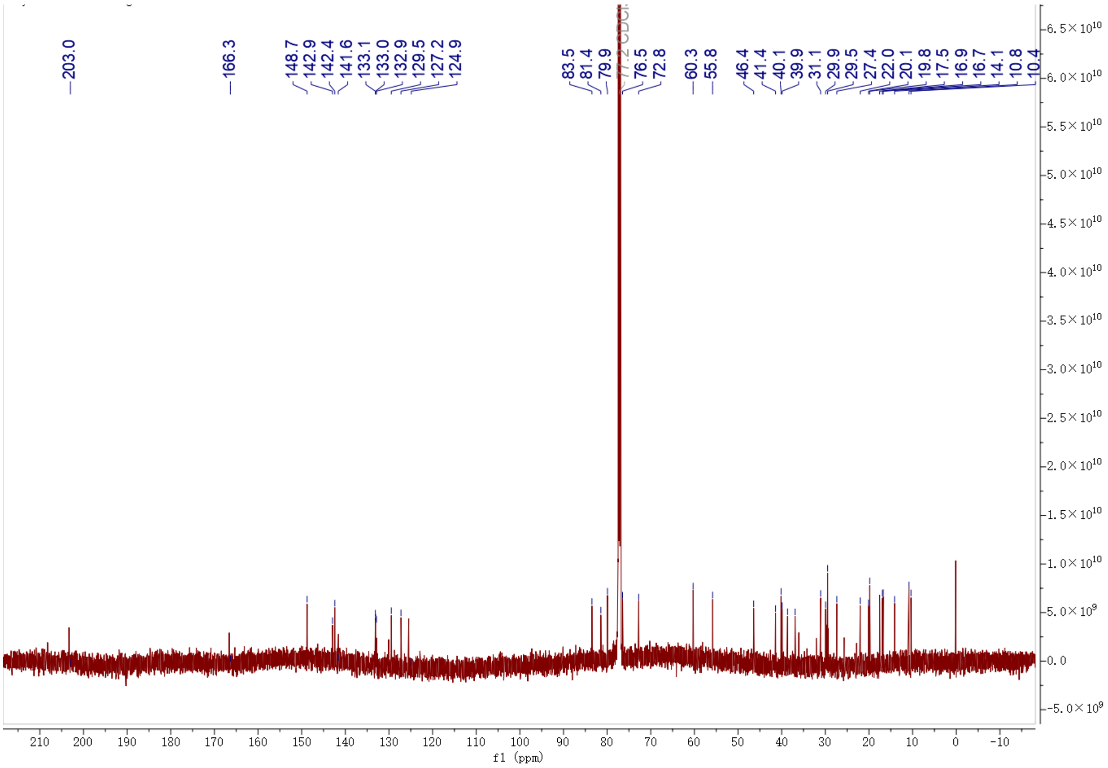

Figure S6. $^{1}H$ NMR (600 MHz, $CDCl_3$) spectrum of bafilomycin.

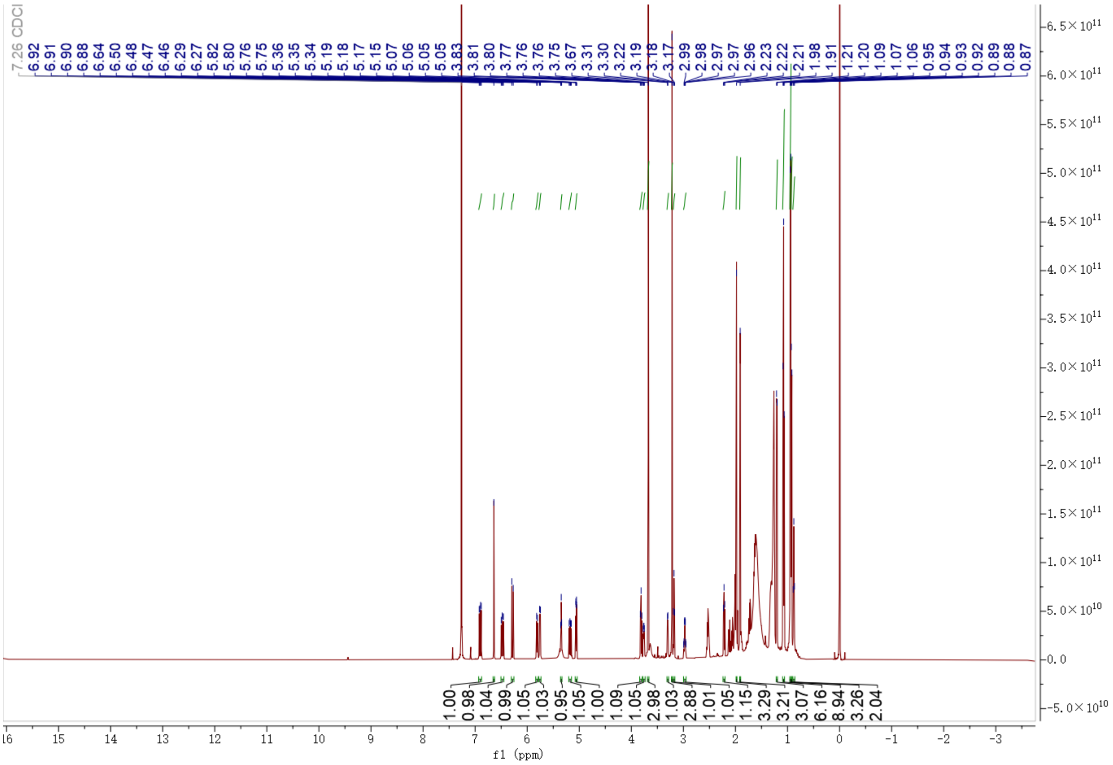

Figure S7. $^{13}C$ NMR (600 MHz, $CDCl_3$) spectrum of 1-Acetyl-β-carboline.

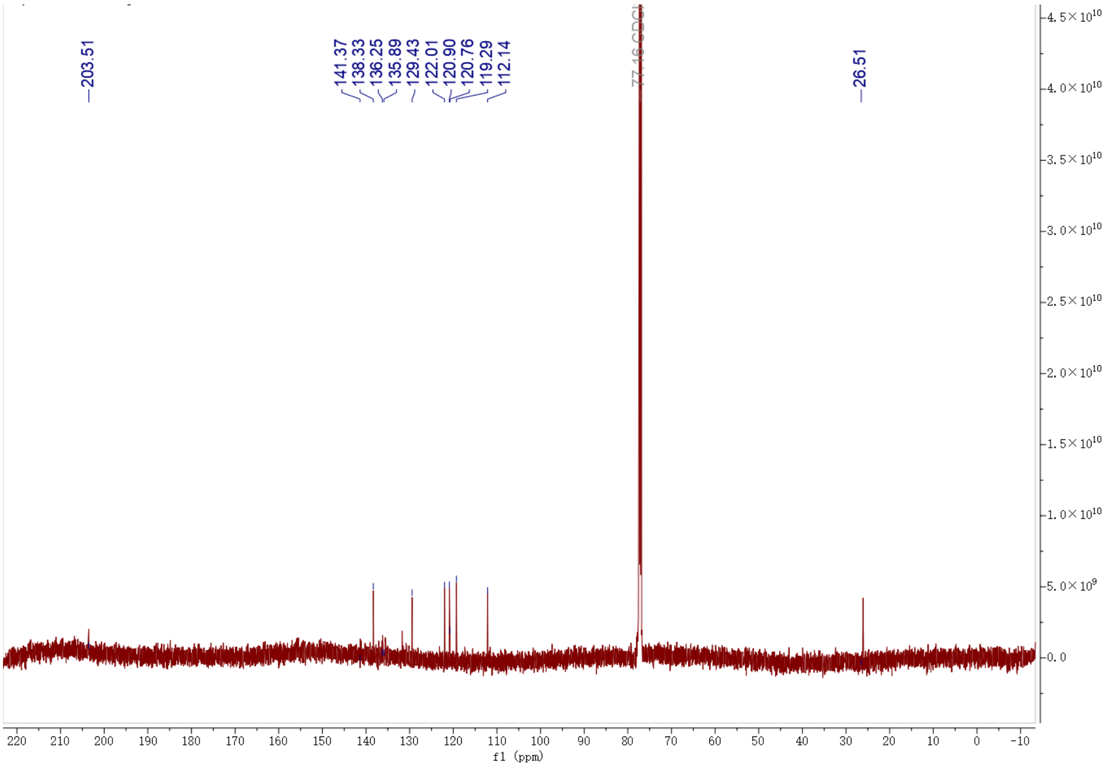

Figure S8. $^{1}$H NMR (600 MHz, $CDCl_3$) spectrum of 1-Acetyl-β-carboline.

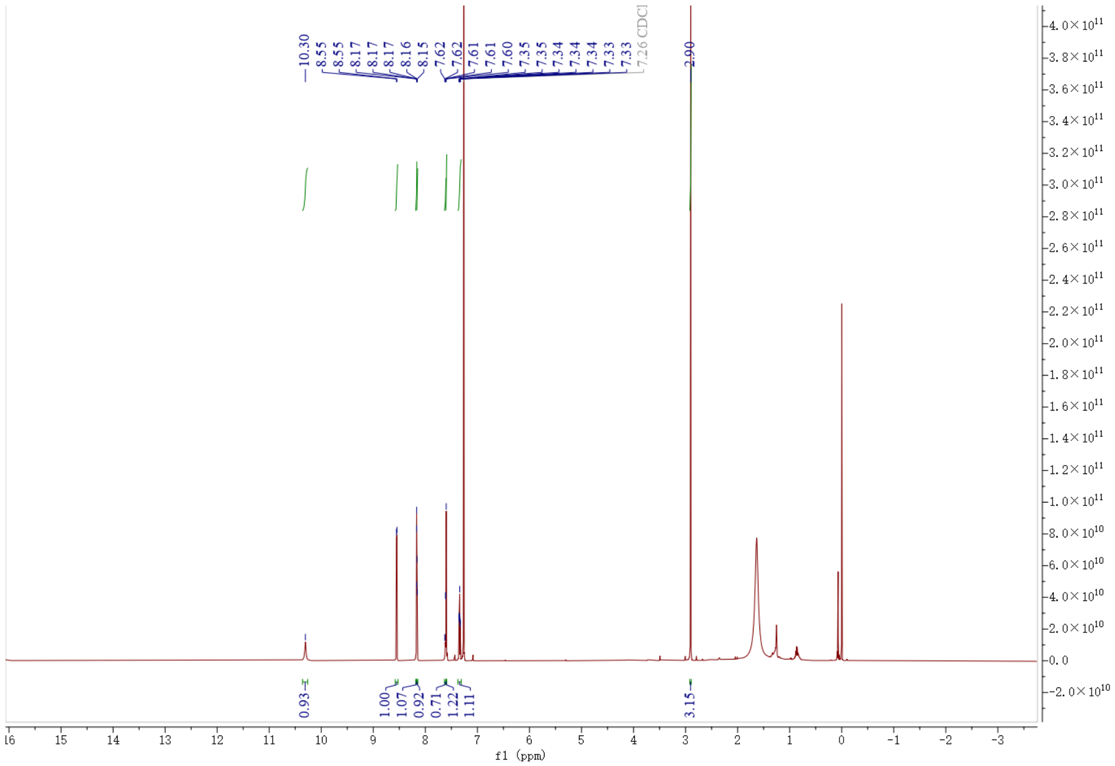

Figure S9. $^{13}C$ NMR (600 MHz, DMSO) spectrum of methyl 4-Hydroxy-3-methoxyacetophenone.

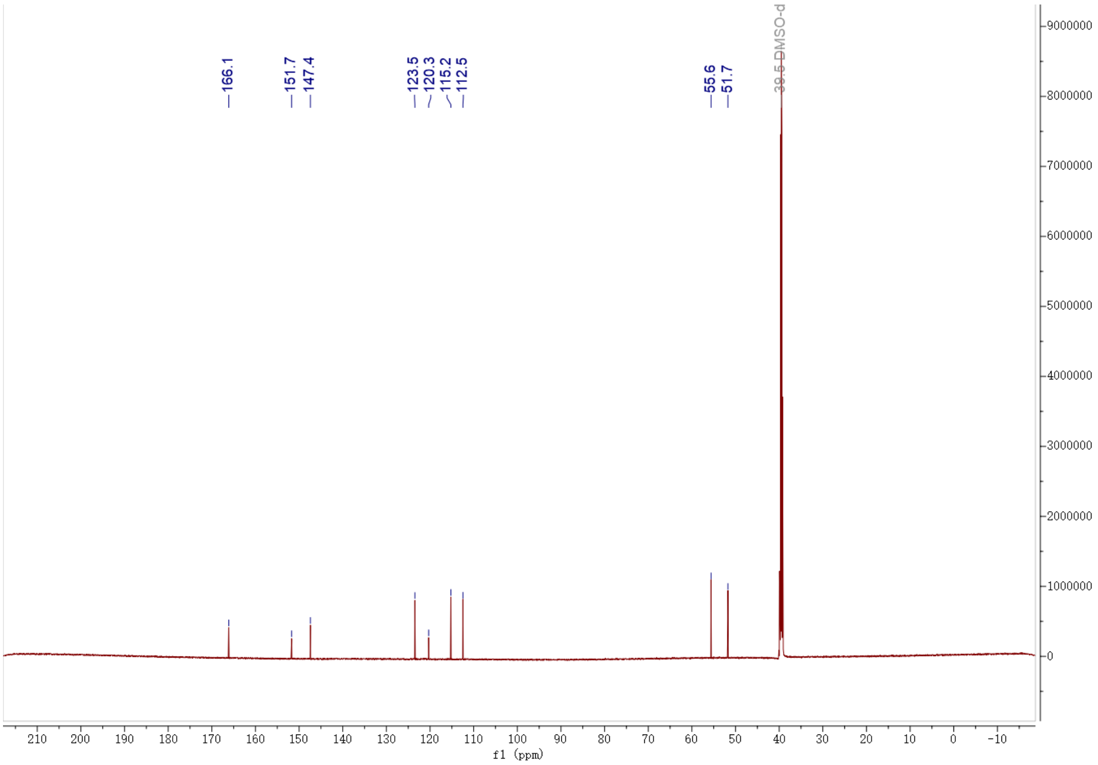

Figure S10. $^{1}H$ NMR(600 MHz, DMSO) spectrum of methyl 4 - Hydroxy - 3-methoxyacetophenone.

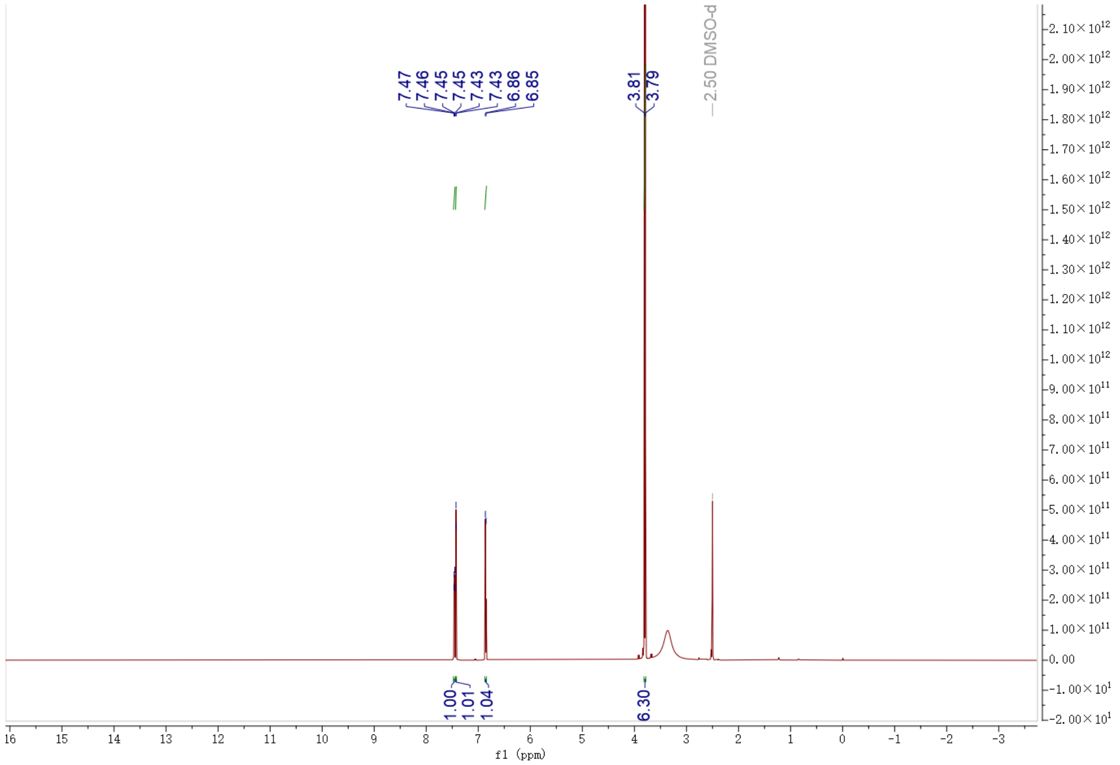

Figure S11. $^{13}$C NMR (600 MHz, DMSO) spectrum of turnagainolide.

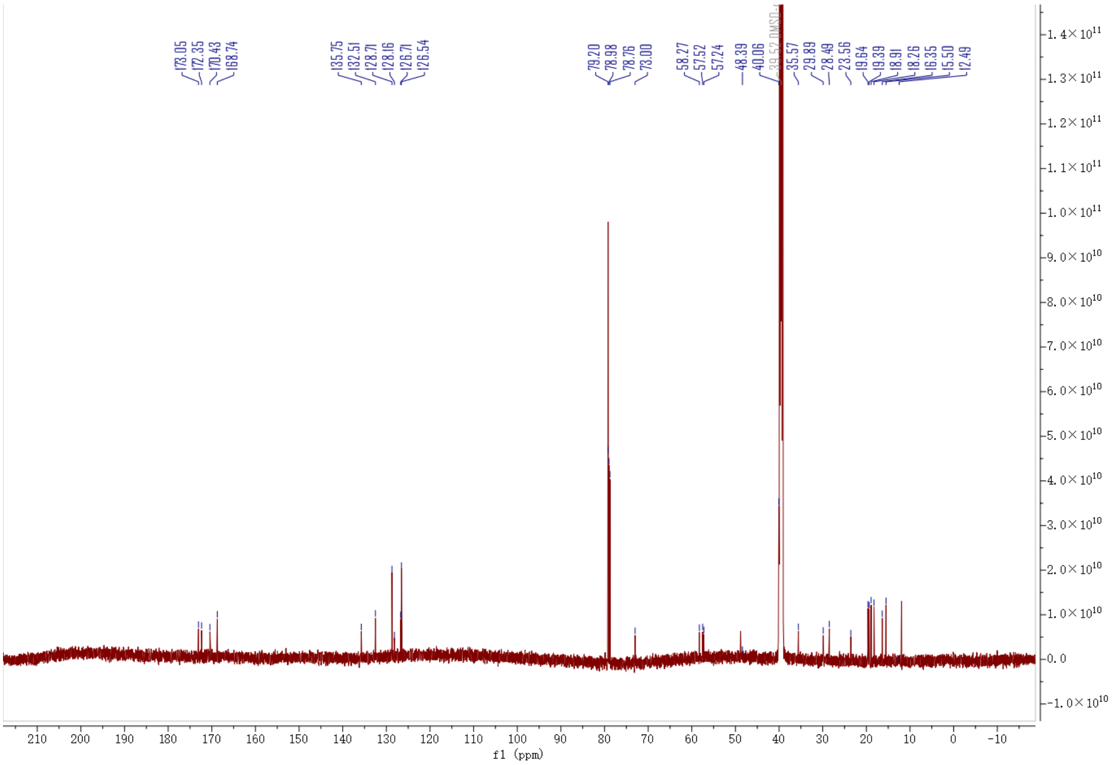

Figure S12. $^{1}$H NMR (600 MHz, DMSO) spectrum of turnagainolide.

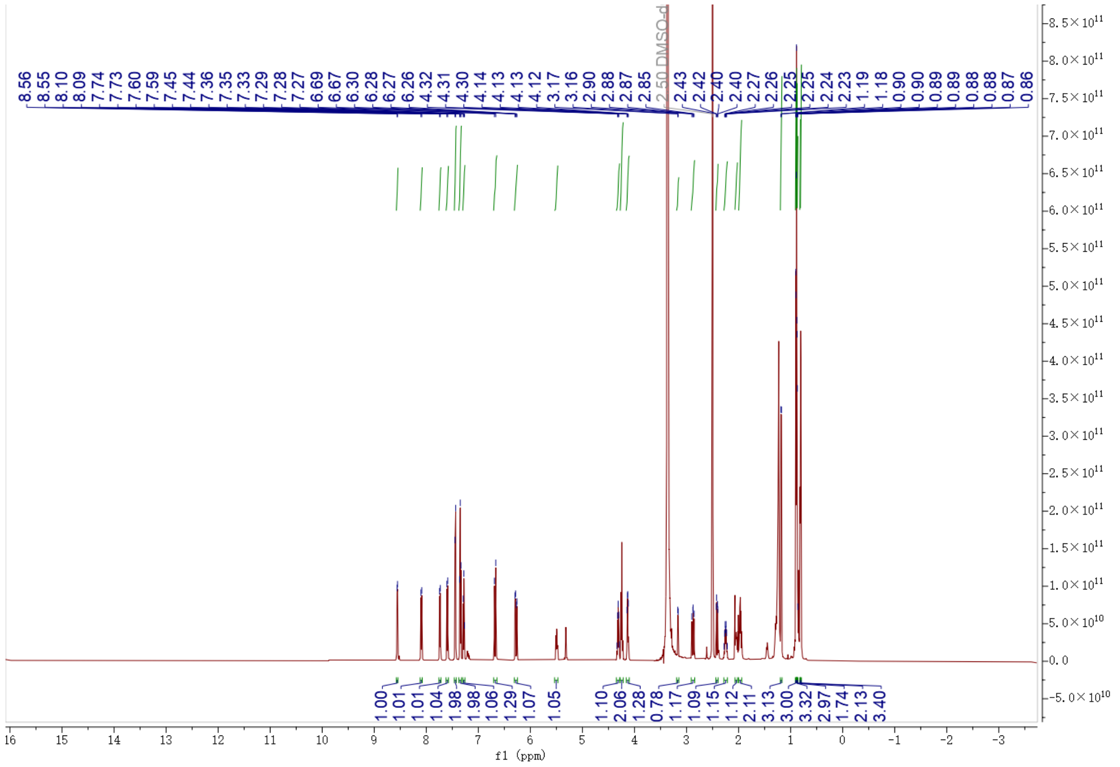

Supplement: Supplementary file 1 [file antibiotics-13-00459-s001.zip › antibiotics-2995627-supplementary.pdf]
